# An R package for SNP marker-based parent-offspring tests

**DOI:** 10.1186/1746-4811-9-44

**Published:** 2013-11-19

**Authors:** Hussein Abdel-Haleem, Pengsheng Ji, H Roger Boerma, Zenglu Li

**Affiliations:** 1 Institute of Plant Breeding, Genetics and Genomics & Center for Applied Genetic Technologies, University of Georgia, 111 Riverbend Rd., Athens, GA 30602, USA; 2 Department of statistics, University of Georgia, 103 Statistics Building, 101 Cedar St., Athens, GA 30602, USA; 3 Georgia Seed Development Commission, 2420 S. Milledge Av., Athens, GA 30605, USA

**Keywords:** SNP markers, Genotyping, Parent-offspring tests

## Abstract

**Background:**

With the advancement of genotyping technologies, whole genome and high-density SNP markers have been widely used for genotyping of mapping populations and for characterization of germplasm lines in many crops. Before conducting SNP data analysis, it is necessary to check the individuals to ensure the integrity of lines for further data analysis.

**Results:**

We have developed an R package to conduct a parent-offspring test of individuals which are genotyped with a fixed set of SNP markers for further genetic studies. The program uses monomorphic SNP loci between parents and their progeny genotypes to calculate the similarity between each offspring and their parents. Based on the similarity of parents and individual offspring, the users can determine the threshold level for the individuals to be included for further data analysis. We used an F_5_-derived soybean population of ‘5601T’ x PI 157440 that was genotyped with 1,536 SNPs to illustrate the procedure and its application.

**Conclusions:**

The R package ‘**ParentOffspring**’ coupled with the available SNP genotyping platforms could be used to detect the possible variants in a specific cross, as well as the potential errors in sample handling and genotyping processes. It can be used in any crop which is genotyped with a fixed set of SNP markers.

## Background

Single nucleotide polymorphism (SNP) genotyping platforms including Invader® assay, single base extension (SBE), oligonucleotide ligation assay (OLA) SNPlex™ system, and the Illumina GoldenGate™ and Infinium™ assays [1] have been developed and widely used to genotype crop plants with a fixed set of SNP markers [2-8]. The SNP data generated from these platforms have been extensively used for genetic and genomic studies including QTL mapping, germplasm characterization, association mapping, and molecular breeding. With a large scale of SNP data available for data analysis, integrity of individuals included in an experiment is very important to ensure the accuracy of the results. For example in a typical QTL mapping population or progeny derived from a cross, variants could result from outcrossing, seed mixing, sampling errors, and many other ways during the population development and sample handling processes. When variants are present, they could distort the experimental results. Although morphological and physiological characteristics can be used to distinguish the variants in a population or seed lot, they are very limited due to availability of phenotypes and accuracy of determination. With the availability of a high density of SNP markers assayed on these advanced genotyping platforms, monomorphic marker loci could be used to detect outcrossing or seed mixture of individuals and possible genotyping errors during sampling and genotyping processes by comparing the SNP alleles of these progeny with their parents’ alleles. In soybean, the Universal Soy Linkage Panel (USLP 1.0) consisting of 1,536 SNPs on the Illumina GoldenGate® Platform has been developed and used for quantitative trait locus (QTL) discovery [6]. Recently, the iSelect Infinium assay which contained over 50,000 SNPs from soybean genome have been also developed [9]. Yan et al. (2009) genotyped 632 inbred maize lines with 1,536 SNPs. Over 200 barley germplasm lines, including European and U.S. breeding materials, were genotyped with 3,072 SNPs on Illumina GoldenGate assays that are available to the barley community [2]. Similarly, 1,536 SNPs on Illumina GoldenGate assays were developed to fingerprint 478 spring and winter wheat lines [8]. A custom-designed Affymetrix array consisting of 44,100 SNPs was used in rice to study the genetic architecture of aluminum tolerance in a bi-parental population and a set of 383 diverse rice accessions [10,11].

With such advanced genotyping technologies, massive amount of SNP data including both polymorphic and monomorphic loci have been generated. Typically, the monomorphic loci are excluded from the data set before further analyses. For example, genotyping a bi-parental population in soybean using an USLP 1.0 panel of 1,536 SNP loci will result in around 1,000 monomorphic loci, which could be used to test if all progeny are truly derived from same parents. Here we developed an R package to calculate the similarity between each offspring and its parents using monomorphic SNP loci. This program can be used by researchers to quality control the progeny and determine which offspring needs to be excluded from further analysis.

## Implementation

To calculate the similarity of each offspring to its parents utilizing the parental monomorphic loci at each monomorphic SNP locus, allele calls in each offspring are assigned with numbers 0, 1, and 2 by comparing the parental genotype. A score of “2” is assigned when an offspring has same alleles to the both parents; a score of “1” indicates that the offspring possesses one parental allele and one non-parental allele; and a score of “0” indicates that the offspring possesses different alleles than their parents. An example of the assignments is presented in Table 1.

**Table 1:** **An example of assigning numerical** (**0**, **1**, **2**) **scores based on the genotypes of progeny and their parents** (**x represents an SNP allele from both parent 1 and parent 2 and y indicates that the SNP allele is not from parent 1 or parent 2**)

**Parent 1**	**Parent 2**	**Progeny**	**Score**
xx	xx	xx	2
xx	xx	xy	1
xx	xx	yy	0

The similarity between an offspring and its parents is calculated based on the all monomorphic loci as follow:

$$S=\left(2a+b\right)\;/\;\left(2a+2b+2c\right)$$

Where S = the similarity between an offspring and its parents, a = the number of markers with a score of 2, and b = the number of markers with a score of 1 and c = the number of markers with a score of 0. The R package can be found in following link (http://cran.r-project.org/web/packages/ParentOffspring/index.html). The output files of this program include both monomorphic and polymorphic SNP marker data set for further analyses.

Theoretically, an offspring should have 100% similarity to its parents. However, due to the possible genotyping errors, outcrossing, seed mixture, and other unknown reasons, similarity of an offspring to its parents could be less than 100%. Reduced similarity will increase the possibility of that offspring being a variant of the specific cross.

## Result and discussion

An F_5_-derived recombinant inbred line soybean population was used as an example to illustrate the procedure and application. The population was developed from a cross of 5601T x PI 157440 at the University of Georgia Plant Sciences Farm, Athens, Georgia. 5601T is a cultivar developed and released by University of Tennessee [12] and PI 157440 was selected as a parent based on its high canopy photosynthetic capacity during the reproductive period [13]. The F_1_ plants were selfed to produce F_2_ plants. Seeds from individual F_2_ plants were advanced to the F_5_ generation using a single-seed descent method [14]. The F_5_ plants were grown at the University of Georgia Plant Sciences Farm and at maturity, individual F_5_ plants were harvested to create 150 F_5:6_ lines. The soybean USLP 1.0 panel of 1,536 SNP markers on the GoldenGate® platform [6] were used to fingerprint these 150 RILs and their parents. The SNP allele calls were performed on the Illumina BeadStation 500G (Illumina, San Diego, CA). The population was evaluated to the bacterial pustule disease reaction (*Xanthomonas campestris pv. Glycines*) under field conditions of UGA Plant Sciences Farm during 2010 in a randomized complete block design with two replications. Based on leaf severity symptoms, the lines were visually rated for bacterial pustule reaction on a plot basis with a scale of 1 to 5, where plots with no symptoms were rated as 1 (Resistant) and plots with severe symptoms as 5 (Susceptible). Associations of the SNP markers with the bacterial pustule rates were tested using single-factor analysis in SAS 9.3 [15].

**Figure 1:**
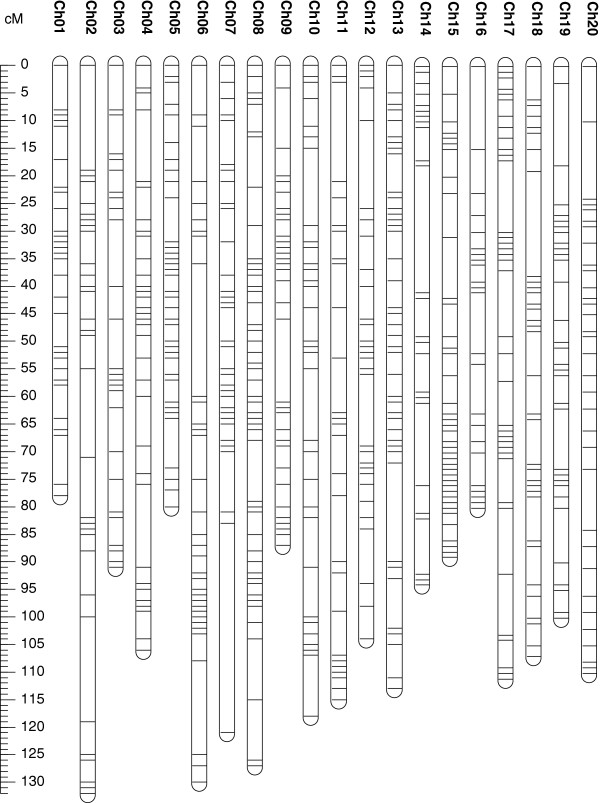
Distribution of monomorphic SNP markers on 20 soybean chromosomes generated from 5601T x PI 157440 population.

Of 1,536 SNP markers in 5601T × PI 157440 population, 542 SNPs (37%) were polymorphic and 938 SNPs monomorphic which accounted for 63% of the total SNP markers. These monomorphic SNPs were distributed eventually on all chromosomes (Figure 1).

**Figure 2:**
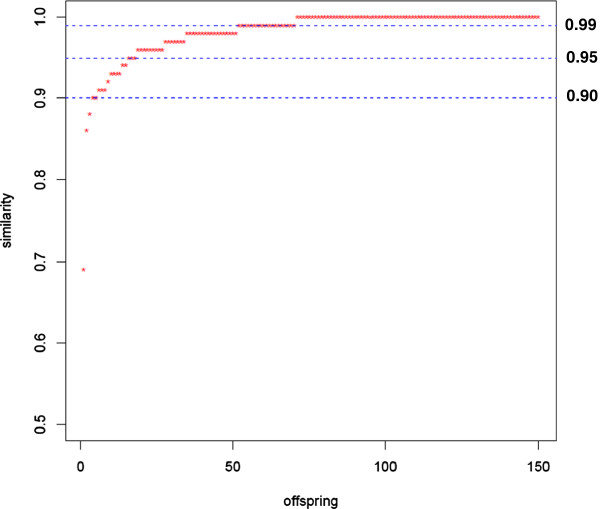
**Distribution of parent-offspring similarity values.**

**Figure 3:**
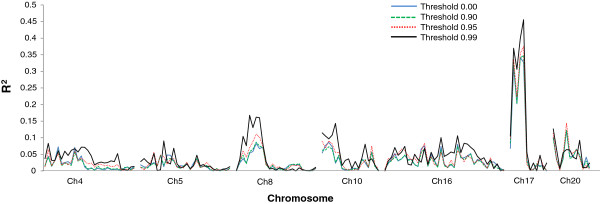
**QTL analysis using single factor analysis for bacterial pustule resistance on seven soybean chromosomes at similarity threshold of 0%, 90%, 95%, and 99%.**

When the similarity threshold was set as 90%, five genotypes were declared as variants; at similarity ratio ≤ 95%, the number of variants was 18, and at more stringent similarity ratio of ≤ 99%, the number of variants reached 70 (Figure 2). The determination of similarity threshold level depends on many factors such as objectives, line types, and genotyping platforms. It is expected that excluding the variants from a population will lead to eliminate the outliners for further genetic analysis. To demonstrate the method, the polymorphic SNP markers data from the 150 RILs of 5601T × PI 157440 population were used to detect QTL associated with resistance to bacterial pustule disease using a single factor analysis approach. Single factor analysis identified a major QTL accounted for 32.7% of the phenotypic variation on chromosome 17 that is in agreement with the reports by Narvel et al. [16]. Based on the similarity threshold levels of 90, 95, and 99% (Figure 3), 5, 18, and 70 lines, respectively, were excluded from the dataset for analysis. The R^2^ for the major QTL on chromosome 17 becomes 34.8, 37.5 and 45.6%, respectively (Figure 3). This indicated that quality control of the offspring using the monomorphic SNP markers could help improving the genetic analyses and thus accuracy of the result. Based on our data, we suggest to use the similarity ratio of 90-95% as a threshold in a study.

## Conclusions

The R package ‘ParentOffspring’ was developed to conduct a parent-offspring test of individuals which are genotyped with a fixed set of SNP markers for further genetic studies. The application of the R package coupled with the available SNP genotyping platforms could be used to detect the possible variants in a specific cross, as well as the potential errors in sample handling and genotyping processes. It can be used in any crop which is genotyped with a fixed set of SNP markers.

## Availability and requirements

**Project name**: ParentOffspring project

**Project home page**: http://cran.r-project.org/web/packages/ParentOffspring/

**Operating system**(**s**): Windows, Mac OS, Linux

**Programming language**: R

**Other requirements**: R version 2.15.1 or higher

**License**: GPL-2 | GPL-3

**Any restrictions to use by non**-**academics**: Non.

## Abbreviations

QTL: Quantitative trait loci; SNP: Single nucleotide polymorphism.

## Competing interests

The authors declare that they have no competing interests.

## Authors’ contributions

HA generated the experimental data, ran the R package and wrote the MS. HA, PJ and ZL developed the procedure, implemented the R package, and evaluated the program. HRB generated the mapping population. All authors participated in the design of the study, edited and approved the final manuscript.
